# Evaluation of Bipolar, Tripolar, and Quadripolar Laplacian Estimates of Electrocardiogram via Concentric Ring Electrodes

**DOI:** 10.3390/s19173780

**Published:** 2019-08-31

**Authors:** Javier Garcia-Casado, Yiyao Ye-Lin, Gema Prats-Boluda, Oleksandr Makeyev

**Affiliations:** 1 Centro de Investigación e Innovación en Bioingeniería, Universitat Politècnica de València, 46022 Valencia, Spain; 2 Department of Mathematics, Diné College, Tsaile, AZ 86556, USA

**Keywords:** electrocardiography, biopotentials, measurement, wearable sensors, concentric ring electrodes, Laplacian, estimation

## Abstract

Surface Laplacian estimates via concentric ring electrodes (CREs) have proven to enhance spatial resolution compared to conventional disc electrodes, which is of great importance for P-wave analysis. In this study, Laplacian estimates for traditional bipolar configuration (BC), two tripolar configurations with linearly decreasing and increasing inter-ring distances (TC_LDIRD_ and TC_LIIRD,_ respectively), and quadripolar configuration (QC) were obtained from cardiac recordings with pentapolar CREs placed at CMV1 and CMV2 positions. Normalized P-wave amplitude (NAP) was computed to assess the contrast to study atrial activity. Signals were of good quality (20–30 dB). Atrial activity was more emphasized at CMV1 (NAP ≃ 0.19–0.24) compared to CMV2 (NAP ≃ 0.08–0.10). Enhanced spatial resolution of TC_LIIRD_ and QC resulted in higher NAP values than BC and TC_LDIRD_. Comparison with simultaneous standard 12-lead ECG proved that Laplacian estimates at CMV1 outperformed all the limb and chest standard leads in the contrast to study P-waves. Clinical recordings with CRE at this position could allow more detailed observation of atrial activity and facilitate the diagnosis of associated pathologies. Furthermore, such recordings would not require additional electrodes on limbs and could be performed wirelessly, so it should also be suitable for ambulatory monitoring, for example, using cardiac Holter monitors.

## 1. Introduction

Cardiovascular disease is the principal cause of morbidity and mortality in developed countries, and in ten years it is expected to become the main cause of death worldwide [1,2]. While mortality associated with cardiovascular disease tends to decrease [3], its costs are expected to increase substantially over the next two decades, mostly due to aging populations [4]. It is estimated that by the year 2035, 131.2 million Americans will suffer from cardiovascular disease with a projected cost of $1.1 trillion [4]. In the European Union, costs associated with cardiovascular disease amount to approximately €169 billion per year [5].

Electrocardiogram (ECG) is the recording of a vital signal extensively used in diagnostics. It provides information not only about the heart rate but also about the electrical conduction in the heart. A wide range of cardiac pathologies can be diagnosed using ECG, such as bundle–branch and atrioventricular blocks, as well as myocardial infarction and dysrhythmias (fibrillations, tachycardias, and bradycardias) [6]. Currently, standard 12-lead ECG does not perform well in the diagnosis of pathologies related to local electrical conduction abnormalities in the heart, such as ventricular ischemia, and especially those related to the atrial activity, with a smaller number of cells involved than in cases of ventricles, such as atrial flutter, often requiring invasive electrophysiology [7]. This is due to the low spatial resolution of bipolar recordings from the conventional disc electrodes commonly used in clinics, which are affected by the blurring effect of the body volume conductor [8]. High spatial resolution non-invasive monitoring systems would be very valuable in the diagnosis of these pathologies. Body surface potential maps, obtained from bioelectric signals recorded from tens of electrodes located on the torso, offer diagnostic information not present in data from 12-lead standard systems that could be crucial in the diagnosis of the aforementioned pathologies [9,10,11]. However, improvement in spatial resolution of the surface potential recordings due to an increase in the number of recording electrodes is limited because of the smearing effect caused by the volume conductor [12]. Body surface Laplacian potential recordings were proposed to overcome this limitation. Surface Laplacian has been shown to be negatively proportional to the two dimensional divergence of the tangential components of the current density on the body surface [13]. This implies that bioelectrical dipoles closest to the recording electrode obtain higher weights than more distant ones, thus improving the dipole source discrimination [14]. Body surface Laplacian maps require placement of a large number of disc electrodes on the chest and application of discrete estimation algorithms. This approach is time consuming and therefore potentially aggravating for clinicians and patients, which hinders its application in clinical practice as a diagnostic tool [15,16,17,18].

Concentric ring electrodes (CREs) in bipolar, quasi-bipolar, and tripolar configurations were proposed to estimate surface Laplacian at each electrode with a linear combination of signals from all of its recording surfaces [12,19]. It has been shown that tripolar CREs provide more accurate estimates of surface Laplacian compared to quasi-bipolar and bipolar CREs while also offering better spatial resolution [19,20]. CREs were first implemented on rigid substrates [20,21,22] and subsequently on flexible substrates such as polyester films [23,24,25] polydimethylsiloxane [26], or textiles [27]. This study builds on a series of studies aiming to improve the accuracy of Laplacian estimation using CREs by optimizing the number of concentric rings [28] and inter-ring distances (distances between the consecutive rings) [29,30]. The main limitation of previous studies [28,29,30] was utilization of the negligible dimensions model (NDM) of a CRE that assumed the radius of the central disc and the widths of concentric rings were negligible. The first step toward a comprehensive finite dimensions model (FDM) of a CRE that includes these additional parameters along with the number of rings and the inter-ring distances included in the NDM has been taken in [31]. Specifically, a Laplacian estimate was derived for a proof of concept tripolar CRE with non-negligible widths of the concentric rings and the radius of the central disc [31]. This was accomplished by representing both concentric rings as clusters of points with specific widths, as opposed to concentric circle representation in NDM [31]. The central disc of the tripolar CRE was also represented by a cluster of points with a specific radius, as opposed to a single point representation in NDM [31]. All the tripolar and quadripolar surface Laplacian estimates derived and assessed in this study are FDM based. Moreover, while NDM based analytic results from [28,29,30] were validated using finite element method modeling, the FDM based surface Laplacian estimates in this paper are assessed using ECG data collected from 20 human subjects via physical CRE prototypes.

Therefore, the first aim of this work was to calculate different estimates of the surface Laplacian using bipolar, tripolar (linearly increasing and decreasing inter-ring distances), and quadripolar CRE configurations of the same size. The second aim was to assess the influence of the configuration on associated spatial resolution and signal quality as well as on the contrast to study the P-wave by assessing various metrics derived from the ECG signals recorded with a CRE sensor node. Comparison with standard 12-lead recordings was also performed.

## 2. Materials and Methods

### 2.1. ECG Recording

Data collection was conducted on twenty volunteers—3 females and 17 males with body mass indices of 25.1 ± 3.2 kg/m^2^ and ages of 36 ± 14.1—at the Health Center of the Universitat Politècnica de València (UPV) when attending routine check-ups. Volunteers were informed of the aim of the study and signed informed consent forms. The study was approved by the UPV Ethics Committee (project identification code P4_20_02_19) and adhered to the Declaration of Helsinki.

Standard 12-lead ECG signals (ECG 8270, Nihon Kohden, Tokyo, Japan) and 6 bipolar concentric ECG (BC-ECG) signals from two wireless CRE sensor nodes were recorded for 5 min for each volunteer. The wireless CRE sensor node consisted of two parts: flexible CRE and electronic circuitry with analog signal processing; and digitization and transmission of three BC-ECG signals [32] as follows:

BC_1_ = V_2_ − V_1_ (1)

BC_2_ = V_3_ − V_1_ (2)

**Figure 1:**
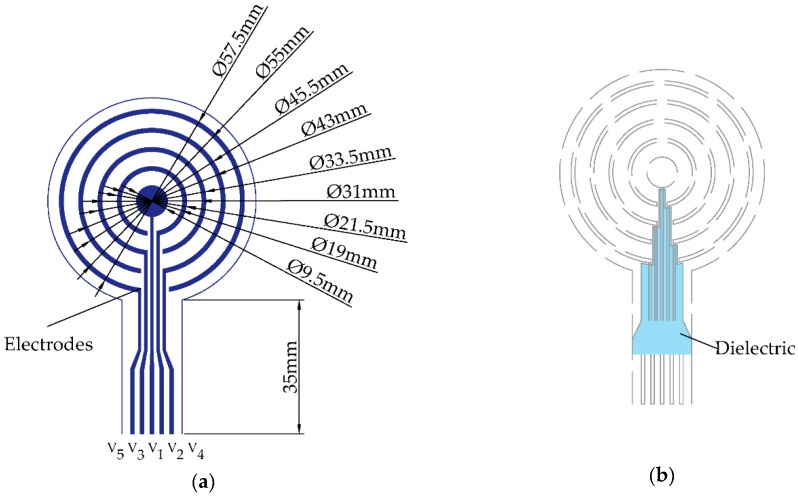
Bilayer design of the concentric ring electrode (CRE): (**a**) recording surfaces including the central disc and open rings along with their dimensions; (**b**) dielectric layer to avoid shorts.

BC_3_ = V_4_ − V_1_ (3)

where V_1_, V_2_, V_3_, and V_4_ are the surface potentials picked up by the central disc and the three open (concentric) rings from the inside out, respectively, as seen in Figure 1. No external reference electrode was used. The outermost CRE ring was connected to an analog ground so as to diminish common mode interference. The node circuitry included 0.3–150 Hz bandpass filtering and 4084 *v*/*v* gain. Sampling rate for bioelectric signals was equal to 500 Hz (with 24-bit resolution), and recorded signals could be either transmitted wirelessly via Bluetooth or stored on a microSD card in real time [32].

**Figure 2:**
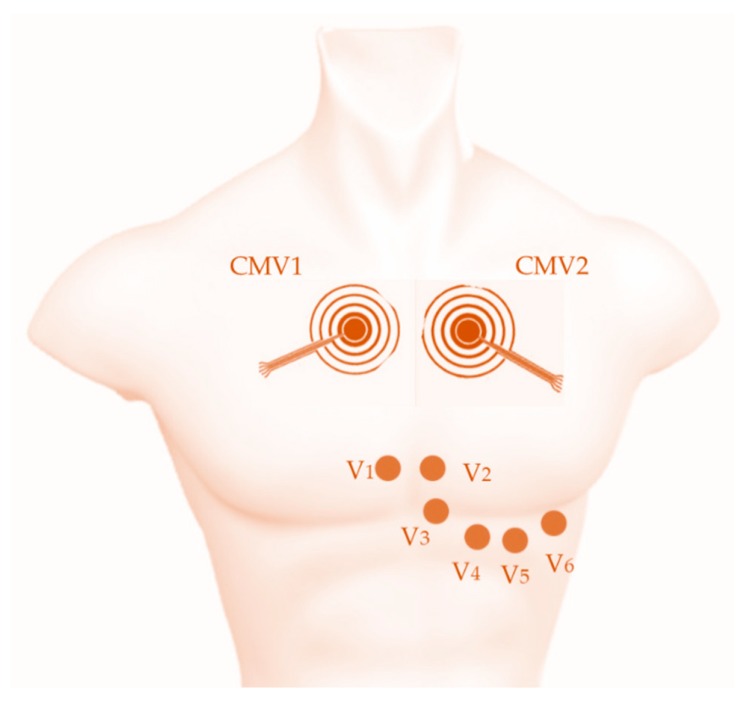
Scheme of the electrode placement for conventional disc electrodes in precordial positions V1, V2, V3, V4, V5, and V6 and two CREs in positions CMV1 (comparable to V1) and CMV2 (comparable to V2) for ECG recording.

Sensor nodes were placed at positions CMV1 (comparable to V1, near the right atria) and CMV2 (similar to V2, near the left atria), as shown in Figure 2, since these were previously determined to be the positions offering the best detectability of cardiac waves in general and of the P-wave in particular [23,33]. To improve skin-contact impedance, the chest area where the electrodes were located was exfoliated using an abrasive gel (Nuprep, Weaver and Company, Aurora, CO, USA) and cleaned with alcohol. This area was also shaved first for male volunteers.

### 2.2. Laplacian Estimates

First, to reduce residual baseline drifts, BC-ECG signals were digitally high-pass filtered (0.1 Hz, zero-phase, fifth-order, Butterworth filter). Next, four FDM based estimates of the surface Laplacian potential corresponding to bipolar (BC), tripolar (with linearly decreasing and increasing inter-ring distances: TC_LDIRD_, TC_LIIRD_), and quadripolar (QC) CRE configurations were computed using filtered BC-ECG signals to be used for subsequent analysis and feature extraction:

BC = BC_3_ (4)

TC_LDIRD_ = BC_2_ − 0.28361·BC_3_ (5)

TC_LIIRD_ = BC_1_ − 0.044389·BC_3_ (6)

QC = BC_1_ − 0.263865·BC_2_ + 0.0304459·BC_3_ (7)

All the CRE configurations had the same size with the third open ring serving as the outer ring. For the case of bipolar configuration (BC) from Equation (4), previous results of Huiskamp were used since the Laplacian estimate in this case was proportional to a single bipolar signal (BC_3_) and not to a linear combination of multiple bipolar signals, as in the case of tripolar and quadripolar configurations [34]. For the cases of linearly decreasing (TC_LDIRD_) and linearly increasing (TC_LIIRD_) inter-ring distance tripolar configurations from Equations (5) and (6), respectively, steps identical to the ones in [31] were used to find the coefficients of the Laplacian estimate using potentials on respective recording surfaces. To compute those potentials, first, the outer radius of the outer ring (radius of the electrode) was set equal to a large arbitrary numeric constant (e.g., 50,000). Next, the radius of the central disc and inner and outer radii of both concentric rings were expressed as integer fractions of this constant based on their actual dimensions from Figure 1a. For example, the radius of the central disc was set equal to 4.8 × 50,000/22.8 ≈ 10,526. Finally, potentials on the recording surfaces were calculated as averages of potentials on all the concentric circles included in them. For example, the potential on the central disc was calculated as the arithmetic mean of 10,527 potentials, including one at the center of the disc and potentials on concentric circles with radii up to 10,526. For the quadripolar configuration (QC) from Equation (7), potentials on the four recording surfaces were calculated in the same way. Next, three bipolar signals (BC_1_, BC_2_, and BC_3_) corresponding to differences of potentials on each of the three concentric rings and on the central disc were linearly combined to cancel out both the fourth and the sixth order truncation terms (as opposed to just the fourth order truncation term being cancelled out in the case of tripolar CRE configurations).

Laplacian estimate coefficients are not unique for FDM, since as is shown in [31], they are a solution of a system of linear equations. Since Laplacian estimate coefficients are determined up to (multiplication by) a constant factor, they were scaled for all four CRE configurations to a unit value of the first coefficient to allow direct comparison.

### 2.3. Data Analysis

ECG fiducial points were identified by detecting the R-wave using Hamilton and Tompkins’ algorithm [35]. The average beat ($\bar{ECG}$) of the Laplacian estimations, BC, TC_LDIRD_, TC_LIIRD_, and QC, was computed in a 60 s window, covering from 250 ms prior to 375 ms after the R-wave.

To compare the signal quality of these Laplacian estimates, the average value of signal-to-noise ratio (SNR) was computed for each recording session.

• Signal-to-noise ratio (SNR): ratio of the peak-to-peak amplitude of the average beat $\bar{ECG}$ and the root mean square (RMS) of the noise during the isoelectric period between beats, the latter being calculated as the RMS value for all the isoelectric periods over the 60 s window.

(8) $$SNR\;\left(dB\right)=20\cdot {log}_{10}\left(\frac{V_{PP}\left(\bar{ECG}\right)}{V_{rms}\left(noise\right)}\right).$$

The absolute signal amplitude of the ECG signal does not provide relevant information as long as the signal quality is good enough. The normalized amplitude of the cardiac waves is relevant since it provides information on the ability to analyze the morphology of each wave for clinical diagnosis. Previous studies have shown than one of the main benefits of Laplacian ECG recording via CREs associated with enhanced spatial resolution is the increase of P-wave contrast assessed by its normalized P-wave amplitude (NAP) [23,26]. Therefore, this metric was computed to compare different Laplacian ECG estimates derived from the CRE and the 12-lead ECG signals:

• NAP: Normalized amplitude of the P-wave with respect to the peak-to-peak amplitude (RS) of the average beat ($\bar{ECG}$).

To assess the variability of NAP for different Laplacian estimates for a given subject, the coefficient of variation (CV_NAP) was computed for each recording session:

• CV_NAP (%): Coefficient of variation of NAP.

(9) $$\mathrm{CV}\_\mathrm{NAP}\;\left(\%\right)\;=\left(\frac{\sigma \left\{{\mathrm{NAP}}_{\mathrm{BC}},\;{\mathrm{NAP}}_{{\mathrm{TC}}_{\mathrm{LIRD}}},\;{\mathrm{NAP}}_{{\mathrm{TC}}_{\mathrm{LDRD}}},\;{\mathrm{NAP}}_{\mathrm{QC}},\right\}}{\mathrm{mean}\left\{{\mathrm{NAP}}_{\mathrm{BC}},\;{\mathrm{NAP}}_{{\mathrm{TC}}_{\mathrm{LIRD}}},\;{\mathrm{NAP}}_{{\mathrm{TC}}_{\mathrm{LDRD}}},\;{\mathrm{NAP}}_{\mathrm{QC}}\right\}}\right)\cdot 100.$$

The Wilcoxon signed rank sum test was performed to assess statistical differences in NAP values for different Laplacian estimates (BC, TC_LDIRD_, TC_LIIRD_, and QC) and those of 12-lead standard recordings. A MANOVA test was performed to compare the metrics for the different Laplacian estimates from positions CMV1 and CMV2.

## 3. Results

### 3.1. Laplacian Estimates of ECG via CRE

**Figure 3:**
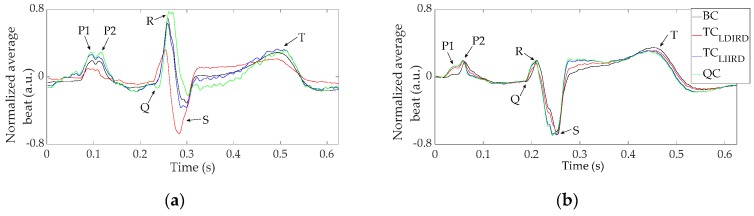
Normalized average beat of the Laplacian estimates via CRE at position CMV1: (**a**) from a volunteer with CV_NAP = 35.0%, (**b**) from a volunteer with CV_NAP = 4.3%. BC: bipolar configuration; TC: tripolar configurations; QC: quadripolar configuration.

Figure 3 shows normalized average beats of the Laplacian estimates (plotted in different colors) via CRE at position CMV1 on two volunteers. Average beats were normalized with respect to their peak-to-peak amplitudes for direct comparison. Traces were clean with slight oscillations after the averaging process. Main cardiac waves (P, Q, R, S, and T) could be clearly observed in all the average beats. As seen in panel (**b**) the morphology barely changed between the different Laplacian estimates while it changed substantially in panel (**a**). A shorter duration of the QR segment and a longer one for RS were observed for TC_LDIRD_. P- and T-waves amplitudes also varied, the smallest being for TC_LDIRD_ in this case. It is noteworthy that two peaks could be observed in the P-wave, probably associated with P1- and P2-waves of left and right atrial activity.

Table 1 shows the results obtained for characteristic metrics derived from the Laplacian estimates via CREs at positions CMV1 and CMV2 for all the volunteers (N = 20). In the case of SNR, the mean values ranged from about 20 dB to 37 dB, showing that the cardiac signal amplitude was between 10 and 70 times greater than that of noise. SNR values from CMV2 were significantly greater than those from CMV1 (*p* < 0.05, MANOVA), indicating the effect of CRE position on the signal quality. It is also noteworthy that, in general, SNR of BC > TC_LDIRD_ > TC_LIIRD_ > QC for both CRE positions (*p* < 0.05, Wilcoxon signed-rank test, except for TC_LIIRD_ vs QC at CMV1). The opposite trend was found in the mean values of NAP related to electrode configuration, with the exception of TC_LIIRD_ and QC at CMV1. Significant differences (*p* < 0.05, Wilcoxon signed-rank test) were only found for BC vs TC_LIIRD_ at both positions, and BC vs QC at CMV2. Cardiac signals recorded at CMV1 resulted in NAP values more than double the values at CMV2 (*p* < 0.05, MANOVA). Mean values of CV_NAP at CMV1 and CMV2 were equal to 26.43% and 20.11%, respectively, indicating that normalized P-wave amplitude varied between different Laplacian estimates for a given volunteer. Standard deviations of CV_NAP were high (20.03% and 11.20%), showing that such variability could change substantially depending on the volunteer. In particular, panel (**a**) of Figure 3 represents a case with high variability of NAP among Laplacian estimates (CV_NAP = 35%), whereas panel (**b**) represents a case with similar morphology and NAP values for different Laplacian estimates (CV_NAP = 4.3%).

### 3.2. Comparison with the Standard 12-Lead ECG

**Figure 4:**
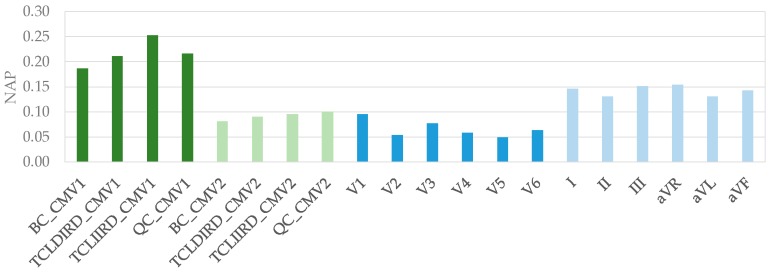
Mean values of NAP for Laplacian estimates via CREs at CMV1 and CMV2 and those for standard 12-lead ECG signals.

Figure 4 shows mean values of NAP obtained for the Laplacian estimates via CREs at CMV1 and CMV2 along with those for standard 12-lead ECG signals. It can be observed that the greatest NAP values were obtained from CRE at CMV1, followed by limb leads (I, II, III, aVR, aVL, and aVF). The highest mean NAP value for standard chest leads was obtained for V1 (NAP = 0.09), which was similar to those from CRE at CMV2.

Statistical comparison of NAP for Laplacian estimates via CREs with those for standard 12-lead ECG is summarized in Table 2. The NAP of signals recorded via CRE at CMV1 was significantly greater than any of the standard 12-lead ECGs (*p* < 0.05, two-sample comparison), with the exception of BC vs lead III. Furthermore, statistical significance was very high (*p* < 0.001) in all the comparisons with standard chest leads and with many of the limb leads. NAP values from CRE signals at CMV2 were significantly greater than those of chest leads, except for V1 and for V3 in some cases, and they were not significantly greater than those of limb leads.

## 4. Discussion

This is the first time that Laplacian quadripolar and LIIRD and LDIRD tripolar estimates were obtained from a physical CRE and that results from real biological signals were analyzed. The experiments were carried out on signals of cardiac origin due to the great relevance and prevalence of cardiovascular diseases and the diagnostic potential associated with the ECG. The PQRST peaks were easily identifiable visually in different Laplacian estimates, and signal quality was good. The signal-to-noise ratio of about 20–30 dB was consistent with other studies on bipolar ECG signals via CREs on textile [27] and polyester [32] substrates.

P-waves, which are associated with atrial activity, are the lowest ECG peaks and the most challenging ones to identify. This is because atrial activation involves fewer cardiac cells than ventricular one. In this context, the position of the recording electrode and its spatial resolution play an important role in providing a good contrast in the monitoring of atrial cardiac activity. Regarding the position, CRE at CMV2 is closer to the heart in general and to the ventricles in particular. On one hand, this is responsible for greater amplitude and quality of signals derived from this site in comparison to CMV1. On the other hand, it also leads to a stronger contribution of ventricular myocytes and therefore larger QRS amplitude with respect to atrial activity (P-wave). At CMV1 the proportional attenuation of ventricular activity was higher than that of the atrial activity, resulting in greater normalized P-wave amplitude. This is in agreement with previously published results on Laplacian estimates with bipolar configuration at these two areas [23,33]. Regarding the spatial resolution, simulation studies have reported that the far-field rejection of tripolar configuration (constant inter-ring distances, TC_CIRD_) is greater than that of bipolar CRE configuration, which is greater than that of a unipolar one (e.g., standard chest leads) [36]. Enhanced spatial selectivity of TC_CIRD_ vs BC has also been experimentally demonstrated in [37]. Moreover, an analytical study validated using finite element method modeling predicted that the error, in descending order, of the Laplacian estimates assessed in this study would be BC > TC_LDIRD_ > TC_LIIRD_ > QC [29]. This is consistent with the experimental results obtained for NAP in this study that showed the poorest contrast of P-wave in BC and increasing NAP values for the other Laplacian estimates at both positions except for the comparison between TC_LIIRD_ and QC at CMV1. It is also consistent with SNR values obtained for different Laplacian estimates i.e., the poorer the spatial resolution, the larger the volume of sensed bioelectric dipoles and the greater the energy of the cardiac signal and the SNR. In the case of cardiac recordings on the chest, there was no significant contribution of bioelectric interferences from other organs. Moreover, the background noise was associated predominantly with the electronic noise in the signal conditioning and acquisition hardware, which was virtually constant regardless of the configuration of the Laplacian estimate. Other studies that compared TC_CIRD_ vs BC Laplacian estimates for EEG [38] and for EMG recordings on the forearm [26] reported higher SNR for TC_CIRD_. In these cases, the greater far-field rejection of TC_CIRD_ yielded a greater attenuation of cardiac interference, which may be responsible for the increased SNR. The robustness of signals from CRE to movements, bad contact, or partial contact during potential clinical operation was not specifically addressed in the present work. Previous works reported similar SNR and saturation percentage of BC ECG records and Mason-Likar Lead-I ECG recordings carried out with commercial wet disk electrodes [39]. BC ECG recorded from textile CREs have been shown to be robust to the artifact’s originating lateral head movement and vertical arm or leg movements, while more sensitive to the one corresponding to deep breathing or laughing [27].

In addition to the type of CRE configuration used for Laplacian estimation, another important factor affecting the spatial resolution is the size of the electrode. A larger electrode size corresponds to worse spatial resolution [32,33,40]. In this study Laplacian estimates via four CRE configurations with the same external diameter of the electrode (45.5 mm) were assessed for direct comparison of the configuration effect. Other studies have reported NAP values of BC recordings at CMV1 of 0.18 [26] and 0.16 [33] with electrodes of external diameter equal to 15 mm and 42 mm, respectively. Despite having used larger electrodes in this study, better spatial resolution of TC_LIIRD_ and QC resulted in higher NAP values (0.25 and 0.22, respectively).

Variability of NAP for different configurations of Laplacian estimates via CREs at a given position was also assessed in this study. Results showed that in some cases, NAP variability was very small (<20% in half of the volunteers) with different estimates being affected by virtually constant scaling factor while preserving similar morphology. Some other cases showed greater variability in NAP and in the morphology and duration of cardiac waves (Figure 3a). This could be related to differences in the physiological constitution of individual subjects and to the relative position and orientation of the heart with respect to the CRE. Previous studies reported similar behavior when studying the influence of ring dimension and, hence, of spatial resolution on BC Laplacian estimates of cardiac signal [32]. Variability of the relative position and orientation of the heart with respect to the CRE for different subjects and enhanced spatial resolution of CREs in comparison with conventional disc electrodes are likely to be responsible for high standard deviation of NAP of a given Laplacian estimate.

Comparison of NAP values for CRE signals with those for a standard 12-lead shows that Laplacian estimates offer better contrast for the study of the P-wave. Specifically, signals from CRE in the CMV1 position outperformed those from all the standard leads, both on limbs and chest. Clinical recordings with CRE at this position could allow more detailed observation of atrial activity and facilitate the diagnosis of associated pathologies. Furthermore, such recordings would not require wires (if a wireless sensor node similar to the one used in this study is adopted) or additional electrodes on limbs, so it should also be suitable for ambulatory monitoring, for example, using cardiac Holter monitors.

## Figures and Tables

**Table 1:** Mean ± standard deviation ^1^ of metrics computed for different Laplacian estimates (BC, TC_LIIRD_, TC_LDIRD_, and QC) at two positions (CMV1 and CMV2).

CRE Position	Laplacian Estimate	SNR (dB)	NAP	CV_NAP (%)
CMV1	BC	29.56 ± 5.62	0.19 ± 0.09	26.43 ± 20.03
	TC_LDIRD_	23.53 ± 5.29	0.21 ± 0.11	
	TC_LIIRD_	19.94 ± 8.48	0.25 ± 0.14	
	QC	19.71 ± 7.38	0.22 ± 0.10	
CMV2	BC	36.99 ± 4.70	0.081 ± 0.034	20.11 ± 11.20
	TC_LDIRD_	34.49 ± 4.56	0.090 ± 0.046	
	TC_LIIRD_	30.66 ± 5.54	0.095 ± 0.041	
	QC	27.44 ± 6.83	0.101 ± 0.048	

^1^*N* = 20 volunteers; NAP: Normalized P-wave amplitude.

**Table 2:** Statistically significant differences ^1^ between NAP values for Laplacian estimates (BC, TC_LIIRD_, TC_LDIRD_, and QC) at the two positions (CMV1 and CMV2) and NAP values for standard 12-lead ECG.

CRE Posit.	Laplac. Estim.	V1	V2	V3	V4	V5	V6	I	II	III	aVR	aVL	aVF
CMV1	BC	***	***	***	***	***	***	*	**		**	*	*
	TC_LDIRD_	***	***	***	***	***	***	***	***	**	***	**	**
	TC_LIIRD_	***	***	***	***	***	***	**	**	*	***	**	**
	QC	***	***	***	***	***	***	**	***	*	***	**	**
CMV2	BC		**		**	***	*						
	TC_LDIRD_		**		**	***	*						
	TC_LIIRD_		**	*	***	***	**						
	QC		***	*	***	***	**						

^1^ * *p* < 0.05, ** *p* < 0.01, *** *p* < 0.001. Wilcoxon signed-rank test, alternative hypothesis: median NAP for Laplacian estimates is greater than that for 12-lead ECG.

## References

[1] Roth G.A., Johnson C., Abajobir A., Abd-Allah F., Abera S.F., Abyu G., Ahmed M., Aksut B., Alam T., Alam K. (2017). Global, Regional, and National Burden of Cardiovascular Diseases for 10 Causes, 1990 to 2015. J. Am. Coll. Cardiol..

[2] Lopez A.D., Mathers C.D., Ezzati M., Jamison D.T., Murray C.J. (2006). Global and regional burden of disease and risk factors, 2001: Systematic analysis of population health data. Lancet.

[3] Bhatnagar P., Wickramasinghe K., Wilkins E., Townsend N. (2016). Trends in the epidemiology of cardiovascular disease in the UK. Heart.

[4] https://healthmetrics.heart.org/cardiovascular-disease-a-costly-burden/.

[5] Leal J., Luengo-Fernández R., Gray A., Petersen S., Rayner M. (2006). Economic burden of cardiovascular diseases in the enlarged European Union. Eur. Heart J..

[6] Malmivuo J. (1995). The Basis of ECG Diagnosis. Bioelectromagnetism Principles and Applications of Bioelectric and Biomagnetic Fields.

[7] Wang Y., Cuculich P.S., Zhang J., Desouza K.A., Vijayakumar R., Chen J., Faddis M.N., Lindsay B.D., Smith T.W., Rudy Y. (2011). Noninvasive Electroanatomic Mapping of Human Ventricular Arrhythmias with Electrocardiographic Imaging. Sci. Transl. Med..

[8] He B., Wu D. (1999). Laplacian electrocardiography. Crit. Rev. Biomed. Eng..

[9] SippensGroenewegen A., Peeters H.A., Jessurun E.R., Linnenbank A.C., Robles de Medina E.O., Lesh M.D., van Hemel N.M. (1998). Body surface mapping during pacing at multiple sites in the human atrium: P-wave morphology of ectopic right atrial activation. Circulation.

[10] Kornreich F., MacLeod R.S., Lux R.L. (2008). Supplemented standard 12-lead electrocardiogram for optimal diagnosis and reconstruction of significant body surface map patterns. J. Electrocardiol..

[11] Fereniec M., Stix G., Kania M., Mroczka T., Maniewski R. (2014). An Analysis of the U-Wave and Its Relation to the T-Wave in Body Surface Potential Maps for Healthy Subjects and MI Patients. Ann. Noninvasive Electrocardiol..

[12] Lian J., Li G., Cheng J., Avitall B., He B. (2002). Body surface Laplacian mapping of atrial depolarization in healthy human subjects. Med. Biol. Eng. Comput..

[13] Wu D., Tsai H.C., He B. (1999). On the estimation of the Laplacian electrocardiogram during ventricular activation. Ann. Biomed. Eng..

[14] He B., Cohen R.J. (1992). Body surface Laplacian mapping of cardiac electrical activity. Am. J. Cardiol..

[15] He B., Cohen R.J. (1992). Body surface Laplacian ECG mapping. IEEE Trans. Biomed. Eng..

[16] He B., Cohen R.J. (1995). Body surface Laplacian electrocardiographic mapping—A review. Crit. Rev. Biomed. Eng..

[17] Umetani K., Okamoto Y., Mashima S., Ono K., Hosaka H., He B. (1998). Body Surface Laplacian Mapping in Patients with Left or Right Ventricular Bundle Branch Block. Pacing Clin. Electrophysiol..

[18] He B., Li G., Lian J. (2002). A spline Laplacian ECG estimator in a realistic geometry volume conductor. IEEE Trans. Biomed. Eng..

[19] Besio W.G., Koka K., Aakula R., Dai W. (2006). Tri-polar concentric ring electrode development for laplacian electroencephalography. IEEE Trans. Biomed. Eng..

[20] Besio W., Aakula R., Koka K., Dai W. (2006). Development of a tri-polar concentric ring electrode for acquiring accurate Laplacian body surface potentials. Ann. Biomed. Eng..

[21] Besio W., Chen T. (2007). Tripolar Laplacian electrocardiogram and moment of activation isochronal mapping. Physiol. Meas..

[22] Lu C.C., Tarjan P.P. (1999). An ultra-high common-mode rejection ratio (CMRR) AC instrumentation amplifier for Laplacian electrocardiographic measurement. Biomed. Instrum. Technol..

[23] Prats-Boluda G., Garcia-Casado J., Martinez-de-Juan J.L., Ye-Lin Y. (2011). Active concentric ring electrode for non-invasive detection of intestinal myoelectric signals. Med. Eng. Phys..

[24] Prats-Boluda G., Ye-Lin Y., Bueno-Barrachina J.M., Rodriguez De Sanabria R., Garcia-Casado J. (2016). Towards the clinical use of concentric electrodes in ECG recordings: Influence of ring dimensions and electrode position. Meas. Sci. Technol..

[25] Zena-Giménez V., Garcia-Casado J., Ye-Lin Y., Garcia-Breijo E., Prats-Boluda G. (2018). A flexible multiring concentric electrode for non-invasive identification of intestinal slow Waves. Sensors.

[26] Ye-Lin Y., Alberola-Rubio J.O., Prats-boluda G., Perales A., Desantes D., Garcia-Casado J. (2015). Feasibility and Analysis of Bipolar Concentric Recording of Electrohysterogram with Flexible Active Electrode. Ann. Biomed. Eng..

[27] Wang K., Parekh U., Pailla T., Garudadri H., Gilja V., Ng T.N. (2017). Stretchable Dry Electrodes with Concentric Ring Geometry for Enhancing Spatial Resolution in Electrophysiology. Adv. Healthc. Mater..

[28] Lidón-Roger J.V., Prats-Boluda G., Ye-Lin Y., Garcia-Casado J., Garcia-Breijo E. (2018). Textile concentric ring electrodes for ECG recording based on screen-printing technology. Sensors.

[29] Makeyev O., Ding Q., Besio W.G. (2016). Improving the accuracy of Laplacian estimation with novel multipolar concentric ring electrodes. Measurement.

[30] Makeyev O., Besio W. (2016). Improving the Accuracy of Laplacian Estimation with Novel Variable Inter-Ring Distances Concentric Ring Electrodes. Sensors.

[31] Makeyev O. (2018). Solving the general inter-ring distances optimization problem for concentric ring electrodes to improve Laplacian estimation. Biomed. Eng. Online.

[32] Makeyev O., Lee C., Besio W.G. Proof of concept Laplacian estimate derived for noninvasive tripolar concentric ring electrode with incorporated radius of the central disc and the widths of the concentric rings. Proceedings of the 2017 39th Annual International Conference of the IEEE Engineering in Medicine and Biology Society (EMBC).

[33] Ye-Lin Y., Bueno-Barrachina J.M., Prats-boluda G., Rodriguez de Sanabria R., Garcia-Casado J. (2017). Wireless sensor node for non-invasive high precision electrocardiographic signal acquisition based on a multi-ring electrode. Meas. J. Int. Meas. Confed..

[34] Prats-Boluda G., Ye-Lin Y., Pradas-Novella F., Garcia-Breijo E., Garcia-Casado J. (2018). Textile Concentric Ring Electrodes: Influence of Position and Electrode Size on Cardiac Activity Monitoring. J. Sens..

[35] Huiskamp G. (1991). Difference formulas for the surface Laplacian on a triangulated surface. J. Comput. Phys..

[36] Hamilton P.S., Tompkins W.J. (1986). Quantitative investigation of QRS detection rules using the MIT/BIH arrhythmia database. IEEE Trans. Biomed. Eng..

[37] Lu C.C., Tarjan P.P. (2002). Pasteless, Active, Concentric Ring Sensors for Directly Obtained Laplacian Cardiac Electrograms. J. Med. Biol. Eng..

[38] Koka K., Besio W.G. (2007). Improvement of spatial selectivity and decrease of mutual information of tri-polar concentric ring electrodes. J. Neurosci. Methods.

[39] Prats-Boluda G., Ye-Lin Y., Barrachina J.B., Senent E., de Sanabria R.R., Garcia-Casado J. (2015). Development of a portable wireless system for bipolar concentric ECG recording. Meas. Sci. Technol..

[40] Kaufer M., Rasquinha L., Tarjan P. Optimization of multi-ring sensing electrode set. Proceedings of the 12th Annual International Conference of the IEEE Engineering in Medicine and Biology Society.

